# Supplementary material for: The neutrophil oxidant hypothiocyanous acid causes a thiol-specific stress response and an oxidative shift of the bacillithiol redox potential in Staphylococcus aureus
Source: Microbiol Spectr. 2023 Nov 6;11(6):e03252-23. doi: 10.1128/spectrum.03252-23 (PMC10715087; doi:10.1128/spectrum.03252-23)
Supplement: Fig. S1 to S5 — Growth curves for phenotype analyses, Brx-roGFP2 biosensor experiments, and aggregation assays. [file spectrum.03252-23-s0001.pdf]

Figure S1

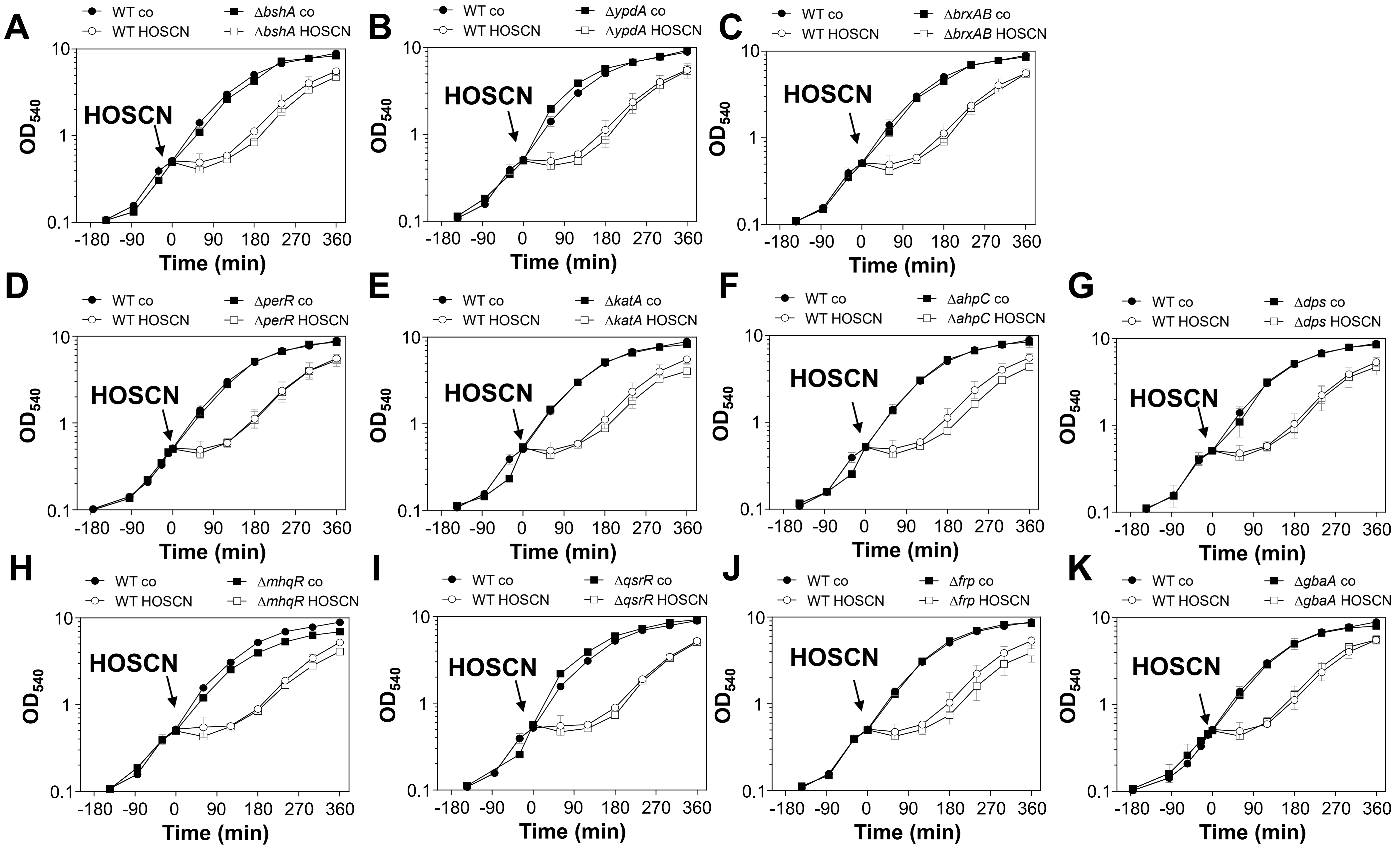

Fig. S1. The antioxidant (PerR, BSH) and electrophile stress systems (QsrR, MhqR, GbaA) do not confer protection against HOSCN stress in *S. aureus* in the presence of MerA. (A-K) The growth of the *S. aureus* COL WT, Δ*bshA*, Δ*ypdA*, Δ*brxAB,* Δ*perR*, Δ*katA*, Δ*ahpC,* Δ*dps*, Δ*qsrR,* Δ*mhqR*, Δ*frp* and Δ*gbaA* mutants were monitored in LB medium after exposure to 176 µM HOSCN at an OD_{540} of 0.5. The graphs show mean values and the errors bars represent the standard deviation (SD) of 3 biological replicate experiments.

Figure S2

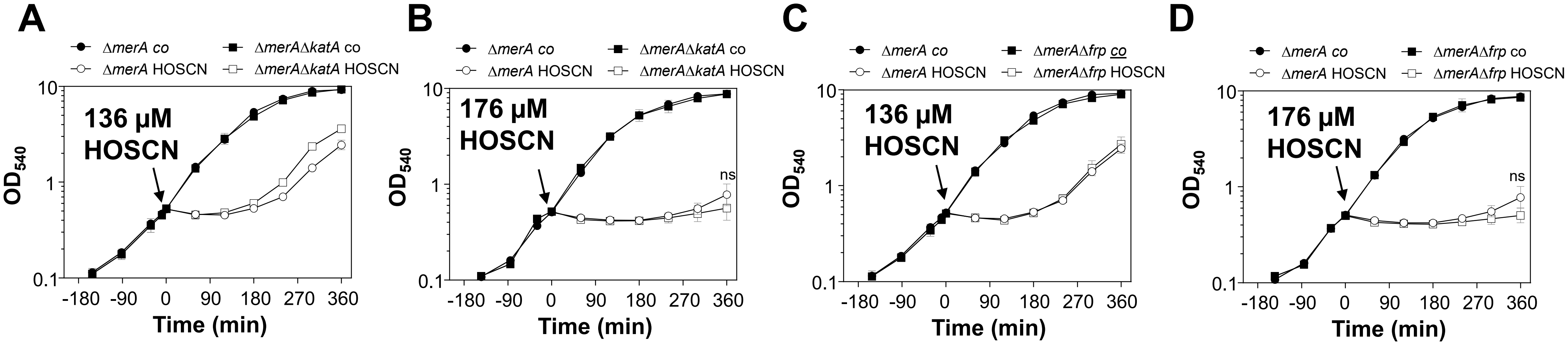

Fig. S2. The *S. aureus* Δ*merA*Δ*katA* (A, B) and Δ*merA*Δ*frp* double mutants (C, D) do not show enhanced sensitivity towards HOSCN stress in comparison to the Δ*merA* mutant. The growth curves of the *S. aureus* COL Δ*merA*, Δ*merA*Δ*katA* and Δ*merA*Δ*frp* mutants were monitored in LB medium after exposure to 136 µM or 176 µM HOSCN at an OD_{540} of 0.5 . Mean values and SD of 2-3 biological replicate experiments are presented.

Figure S3

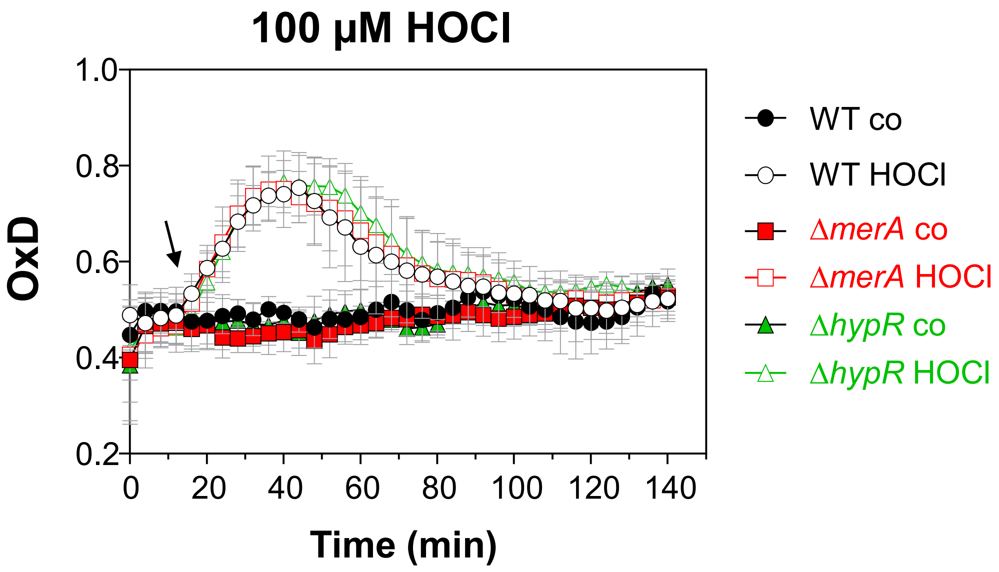

Fig. S3. MerA does not have an impact on the oxidative shift in E_{BSH} and the recovery after sublethal HOCl stress as revealed by the Brx-roGFP2 biosensor. *S. aureus* COL WT, Δ*merA* and Δ*hypR* mutant strains expressing the Brx-roGFP2 biosensor were treated with 100 µM HOCl and the biosensor oxidation degree (OxD) was monitored using the CLARIOSTAR microplate reader. The Brx-roGFP2 biosensor showed a similar oxidative shift of E_{BSH} upon HOCl stress and in the recovery of reduced E_{BSH} in the WT, Δ*merA* and Δ*hypR* mutants. Mean values and SD of 3–4 biological replicates are presented.

Figure S4

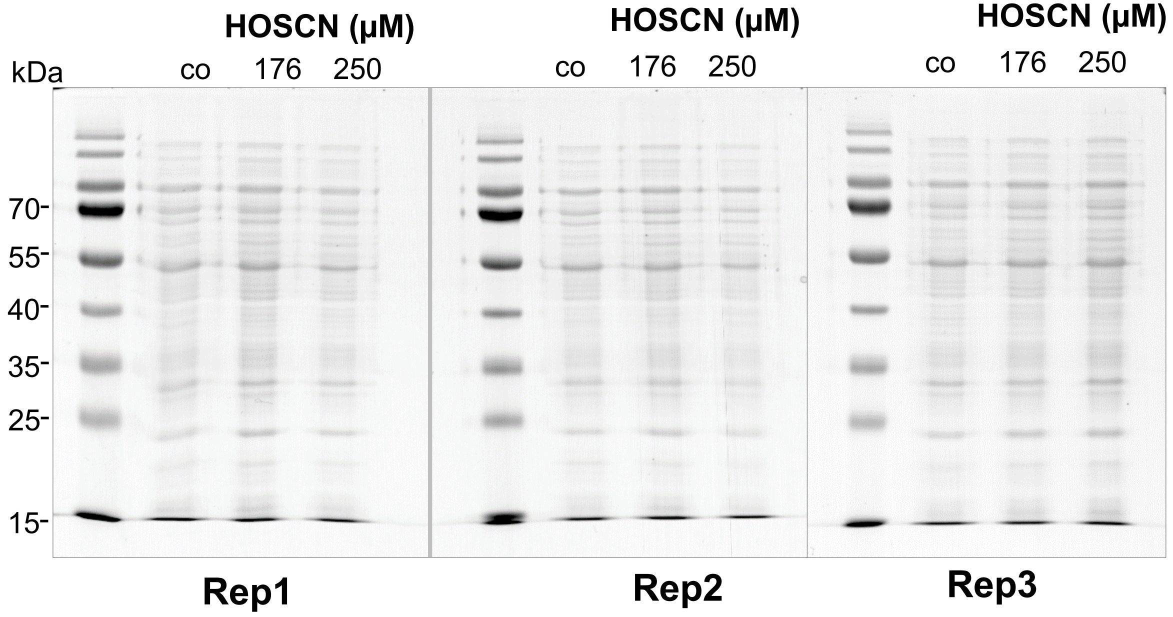

Fig. S4. HOSCN stress does not lead to increased protein aggregation. Intracellular protein aggregates were isolated from *S. aureus* COL WT cells grown in LB before (co) and after the exposure to 176 and 250 µM HOSCN stress at an OD_{540} of 0.5, as described in the Methods section. The results are from 3 biological replicates (Rep1-3).

Figure S5

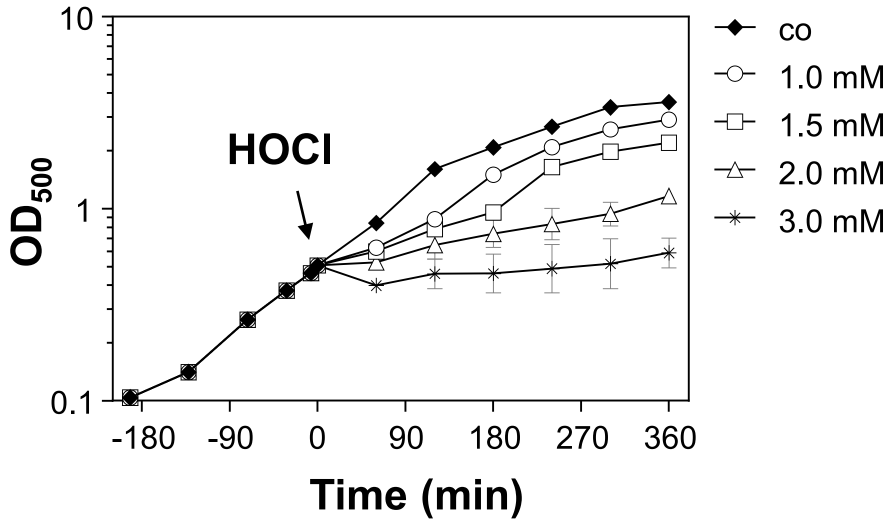

Fig. S5. Growth of *S. aureus* COL in RPMI medium after exposure to different doses of HOCl. *S. aureus* COL was cultivated in RPMI to an OD_{500} of 0.5, and exposed to 1-3 mM HOCl to monitor the effect of HOCl on the growth. Mean values and SD of 3 biological replicates are presented.
