# Supplementary material for: The neutrophil oxidant hypothiocyanous acid causes a thiol-specific stress response and an oxidative shift of the bacillithiol redox potential in Staphylococcus aureus
Source: Microbiol Spectr. 2023 Nov 6;11(6):e03252-23. doi: 10.1128/spectrum.03252-23 (PMC10715087; doi:10.1128/spectrum.03252-23)
Supplement: Tables S5 and S6 — Bacterial strains, phages, plasmids, and oligonucleotide primers. [file spectrum.03252-23-s0003.pdf]

Table S5. Bacterial strains, phages and plasmids.

Strain	Description	Reference
Staphylococcus aureus		
RN4220	restriction negative strain/MSSA cloning intermediate derived from 8325-4	(1)
COL	archaic HA-MRSA strain	(2)
COL-Δ*hypR*	COL *hypR* deletion mutant	(3)
COL-Δ*hypR*-pRB473-*hypR*	COL *hypR* deletion mutant complemented with pRB473-*hypR*	(3)
COL-Δ*merA*	COL *merA* deletion mutant	(3)
COL-Δ*merA*-pRB473-*merA*	COL *merA* deletion mutant complemented with pRB473-*merA*	(3)
COL-pRB473-*brx-roGFP2*	COL expressing the Brx-roGFP2 biosensor	(4)
COL-Δ*hypR*-pRB473-*brx-roGFP2*	COL Δ*hypR* expressing the Brx-roGFP2 biosensor	This study
COL-Δ*merA*-pRB473-*brx-roGFP2*	COL Δ*merA* expressing the Brx-roGFP2 biosensor	This study
COL-Δ*katA*	COL *katA* deletion mutant	(5)
COL-Δ*ahpC*	COL *ahpC* deletion mutant	(6)
COL-Δ*ahpC*Δ*katA*	COL *ahpC katA* double deletion mutant	(6)
COL-Δ*perR*	COL *perR* deletion mutant	(6)
COL-Δ*bshA*	COL *bshA* deletion mutant	(5)
COL-Δ*brxAB*	COL *brxA brxB* double deletion mutant	(7)
COL-Δ*ypdA*	COL *ypdA* deletion mutant	(7)
COL-Δ*qsrR*	COL *qsrR* deletion mutant	(8)
COL-Δ*mhqR*	COL *mhqR* deletion mutant	(8)
COL-Δ*frp*	COL *frp* deletion mutant	This study
COL-Δ*dps*	COL *dps* deletion mutant	This study
COL-Δ*gbaA*	COL *gbaA* deletion mutant	(9)
COL-Δ*merA*Δ*bshA*	COL *merA bshA* double deletion mutant	This study
COL-Δ*merA*Δ*katA*	COL *merA katA* double deletion mutant	This study
COL-Δ*merA*Δ*frp*	COL *merA frp* double deletion mutant	This study
Staphylococcus phage 81		(10)
Escherichia coli		
DH5α	F-φ80dlacZ Δ(lacZYA-argF) U169 deoRsupE44ΔlacU169 (f80lacZDM15) hsdR17 recA1 endA1 (rk- mk+) supE44gyrA96 thi-1 gyrA69 relA1	(11)
**Plasmids**		
pMAD	Temperature-sensitive shuttle vector for gene replacement in *S. aureus* Amp^R, Em^R	(12)
pMAD-Δ*frp*	pMAD plasmid carrying up- and downstream flanking regions of *frp* gene	This study
pMAD-Δ*dps*	pMAD plasmid carrying up- and downstream flanking regions of *dps* gene	This study
pRB473	pRB373-derivative, *E. coli*/ *S. aureus* shuttle vector, containing xylose-inducible P_{Xyl} promoter Amp^R, Cm^R	(13, 14)
pRB473-*brx-roGFP2*	pRB473 expressing *brx-roGFP2* under P_{Xyl}	(4)

^{R}: resistant, Amp: ampicillin, Cm: chloramphenicol, Em: erythromycin

Table S6. Oligonucleotide sequences

Primer name	Sequence (5' to 3')
pMAD-frp-f1-for-BglII	CGCAGATCTAGTACGTTGTGCAATTTGTTTAAT
pMAD-frp-f1-rev	GGTCCAACCCATTCAATAACATATGCATCCATAATTGTTTGATTCA
pMAD-frp-f2-for	TGAATCAAACAATTATGGATGCATATGTTATTGAATGGGTTGGACC
pMAD-frp-f2-rev-SalI	CCAGTCGACACTTTAGAAGTTGATAGCCAAAC
pMAD-dps-for-BglII	CGCAGATCTTGGCCAACAATTTTTTAAACAAC
pMAD-dps-f1-rev	ACTTATCTGTAGATTAGCTTAAGGATTACTCATATTTAATACACTCC
pMAD-dps-f2-for	GGAGTGTATTAAATATGAGTAATCCTTAAGCTAATCTACAGATAAGT
pMAD-dps-rev-SalI	CCAGTCGACCTGCATCAACTAATTCAAATTATG
NB-frp-for	GCATTTCATTTCAGACATGCGA
NB-frp-rev	CTAATACGACTCACTATAGGGAGATTTCGGTGGCTCTTGTTGTC
NB-hchA-for	AAGTAAGCAACCAACGCCAG
NB-hchA-rev	CTAATACGACTCACTATAGGGAGAAACCATTTCAAGCGTCCAGG

Restriction sites are underlined

Supplementary References

1. Kreiswirth BN, Lofdahl S, Betley MJ, O'Reilly M, Schlievert PM, Bergdoll MS, Novick RP. 1983. The toxic shock syndrome exotoxin structural gene is not detectably transmitted by a prophage. Nature 305:709-12.

2. Shafer WM, Iandolo JJ. 1979. Genetics of staphylococcal enterotoxin B in methicillin-resistant isolates of *Staphylococcus aureus*. Infect Immun 25:902-11.

3. Loi VV, Busche T, Tedin K, Bernhardt J, Wollenhaupt J, Huyen NTT, Weise C, Kalinowski J, Wahl MC, Fulde M, Antelmann H. 2018. Redox-sensing under hypochlorite stress and infection conditions by the Rrf2-family repressor HypR in *Staphylococcus aureus*. Antioxid Redox Signal 29:615-636.

4. Loi VV, Harms M, Müller M, Huyen NTT, Hamilton CJ, Hochgräfe F, Pane-Farre J, Antelmann H. 2017. Real-time imaging of the bacillithiol redox potential in the human pathogen *Staphylococcus aureus* using a genetically encoded bacilliredoxin-fused redox biosensor. Antioxid Redox Signal 26:835-848.

5. Linzner N, Fritsch VN, Busche T, Tung QN, Loi VV, Bernhardt J, Kalinowski J, Antelmann H. 2020. The plant-derived naphthoquinone lapachol causes an oxidative stress response in *Staphylococcus aureus*. Free Radic Biol Med 158:126-136.

6. Linzner N, Loi VV, Antelmann H. 2022. The catalase KatA contributes to microaerophilic H_2O_2 priming to acquire an improved oxidative stress resistance in *Staphylococcus aureus*. Antioxidants (Basel) 11.

7. Linzner N, Loi VV, Fritsch VN, Tung QN, Stenzel S, Wirtz M, Hell R, Hamilton CJ, Tedin K, Fulde M, Antelmann H. 2019. *Staphylococcus aureus* uses the bacilliredoxin (BrxAB)/bacillithiol disulfide reductase (YpdA) redox pathway to defend against oxidative stress under infections. Front Microbiol 10:1355.

8. Fritsch VN, Loi VV, Busche T, Sommer A, Tedin K, Nürnberg DJ, Kalinowski J, Bernhardt J, Fulde M, Antelmann H. 2019. The MarR-type repressor MhqR confers quinone and antimicrobial resistance in *Staphylococcus aureus*. Antioxid Redox Signal 31:1235-1252.

9. Van Loi V, Busche T, Fritsch VN, Weise C, Gruhlke MCH, Slusarenko AJ, Kalinowski J, Antelmann H. 2021. The two-Cys-type TetR repressor GbaA confers resistance under disulfide and electrophile stress in *Staphylococcus aureus*. Free Radic Biol Med 177:120-131.

10. Rosenblum ED, Tyrone S. 1964. Serology, density, and morphology of staphylococcal phages. J Bacteriol 88:1737-42.

11. Studier FW, Moffatt BA. 1986. Use of bacteriophage T7 RNA polymerase to direct selective high-level expression of cloned genes. J Mol Biol 189:113-30.

12. Arnaud M, Chastanet A, Debarbouille M. 2004. New vector for efficient allelic replacement in naturally nontransformable, low-GC-content, gram-positive bacteria. Appl Environ Microbiol 70:6887-91.

13. Brückner R, Wagner E, Götz F. 1993. Characterization of a sucrase gene from *Staphylococcus xylosus*. J Bacteriol 175:851-7.

14. Pöther DC, Gierok P, Harms M, Mostertz J, Hochgräfe F, Antelmann H, Hamilton CJ, Borovok I, Lalk M, Aharonowitz Y, Hecker M. 2013. Distribution and infection-related functions of bacillithiol in *Staphylococcus aureus*. Int J Med Microbiol 303:114-23.
